# Integration of Single‐Cell RNA Sequencing Data and Bulk Sequencing Data to Characterise the CD8+ T‐Cell Exhaustion Mediated Immune Microenvironment in CRC

**DOI:** 10.1111/jcmm.70556

**Published:** 2025-05-12

**Authors:** Xiao‐Hua Ling, Gang Chen, Nan‐Nan Liu, Wen‐Xin Xu, Ming‐Feng Ding

**Affiliations:** ^1^ Department of Gastroenterology The Fourth Affiliated Hospital of Harbin Medical University Harbin People's Republic of China; ^2^ Department of General Surgery The Fourth Hospital of Harbin Medical University Harbin Heilongjiang People's Republic of China; ^3^ Department of Intensive Care Unit The Fourth Affiliated Hospital of Harbin Medical University Harbin Heilongjiang People's Republic of China

**Keywords:** CD8+ T cells, colorectal cancer, prognostic model, scRNA‐seq

## Abstract

CD8+ T cells are crucial for the anti‐tumour immune response, and their exhaustion contributes to poor prognosis and limited immunotherapy efficacy in colorectal cancer (CRC). In this study, we examined the immune microenvironment of CRC by integrating single‐cell RNA sequencing (scRNA‐seq) and bulk sequencing data. T‐cell subtypes in tumour tissues were analysed using CellMarker 2.0 and scType, and an intercellular communication network was constructed through CellChat. Our analysis revealed that exhausted CD8+ T cells exhibit strong interactions with epithelial cells, primarily via the MIF‐(CD74 + CXCR4), MIF‐(CD74 + CD44) and CD99‐CD99 pathways. Based on CD8+ T‐cell exhaustion markers, we developed a prognostic model using XGBoost, which demonstrated promising predictive capabilities for CRC prognosis and immunotherapy response. Functional assays showed that MIF knock‐down significantly inhibited CRC cell proliferation and invasion. Our findings suggest that MIF and CD99 are key regulators of CD8+ T‐cell exhaustion in CRC. This study provides novel insights into the mechanisms underlying T‐cell exhaustion in CRC and offers potential biomarkers for improving immunotherapy outcomes.

## 1 Introduction

Colorectal cancer (CRC) is a malignancy with a high morbidity and mortality rate worldwide [1]. Recently, tumour immunotherapy has attracted much of the interest in recent years as a novel therapeutic approach [2]. CD8+ T cells are critical effector T cells that can recognise and kill tumour cells in tumour immunosurveillance [3]. The process of CD8+ T cells in tumour tissue can be activated by immunotherapy, which in turn enhances immunotherapy responsiveness [4]. Studies have shown that the infiltration level of CD8+ T cells in tumour tissues such as lung cancer, CRC and melanoma is strongly correlated with prognosis and immunotherapy responsiveness [5]. However, prolonged exposure to the suppressive tumour microenvironment can lead to functional impairment and CD8+ T‐cell exhaustion [6, 7]. Dolina's work has shown that the exhausted state of T cells leads to loss of function in terms of cell proliferation, effector secretion and killing capacity [8]. Beltra et al. showed that there are pathogenetic relationships and differentiation trajectories between different CD8+ T‐cell depletion sub‐populations and that interventions can be targeted at the different subsets of exhaustion in the treatment process [9]. It suggests that the development of CD8+ T‐cell exhaustion is a complex multifactorial process. Reversing CD8+ T‐cell exhaustion has become an attractive research direction to promote the responsiveness of immunotherapy [10]. Therefore, it is essential to further investigate the biological characteristics and mechanisms of CD8+ T‐cell exhaustion.

Single‐cell RNA sequencing (scRNA‐seq) and bulk sequencing have been widely applied in the field of investigating the immune microenvironment of tumours [11]. scRNA‐seq reveals the gene expression profile and intercellular heterogeneity of individual cells, uncovering the expression profiles and transcriptome heterogeneity of different cell types [12]. scRNA‐seq allows researchers to more precisely delineate intercellular heterogeneity and linkages and increase the sensitivity of identifying rare cell types [13]. Compared to scRNA‐seq, bulk sequencing is superior in terms of gene expression coverage and accuracy, allowing detection of less expressed genes. Bulk sequencing provides lower technical noise and sequencing error by analysing average expression [14]. ScRNA‐seq and bulk sequencing have their own limitations and advantages [15, 16]. By integrating these two technologies for analysis, a more accurate and precise distribution, phenotype and functional status of tumour immune cells can be obtained, providing an important reference for the development of immunotherapy strategies.

In this study, we characterised the detailed landscape of the tumour immune microenvironment in CRC by integrating the bulk sequencing and single‐cell sequencing data. We focused our study on CD8+ T cells from the perspectives of pathway enrichment analysis, cell differentiation trajectory and intercellular communication, and provided insights into the mechanism of CD8+ T‐cell exhaustion phenotype generation. In addition, 34 markers associated with CD8+ T‐cell exhaustion were screened. We integrated the bulk sequencing data results to construct a predictive model for prognosis and immunotherapy responsiveness. After that, we performed preliminary validation on the bulk sequencing dataset of the immunotherapy cohort. This study provides a preliminary investigation of the mechanisms that generate CD8+ T‐cell exhaustion in CRC tumour tissue and stratifies patient prognosis and immunotherapy responsiveness based on phenotypic markers of exhaustion. Our study is dedicated to promoting the development of precision therapy and provides a degree of theoretical basis for subsequent research on immunotherapy of CRC tumours.

## 2 Methods and Materials

### 2.1 Bulk Data Collection

Transcriptome data and clinical information of CRC patients [including colon adenocarcinoma (COAD) and rectum adenocarcinoma (READ)] of The Cancer Genome Atlas (TCGA) were accessed from UCSC Xena (https://xenabrowser.net/). Gene expression and clinical information from 68 metastatic colorectal cancer patients receiving chemotherapy (GSE72968) were downloaded from Gene Expression Omnibus (GEO) (http://www.ncbi.nlm.nih.gov/geo).

Transcriptomic and clinical information of 91 melanoma patients treated with anti‐PD‐1 monotherapy was originally published by Gide et al.'s [17] and later utilized by Lee et al.; we obtained the data directly from Lee et al.’s work [18] (https://zenodo.org/record/4661265#.ZB2PBXZBxGM).

Raw sequencing data and clinical data of 153 melanoma patients who received anti‐PD‐1 treatment from Van Allen et al.'s study were originally provided in dbGaP (https://www.ncbi.nlm.nih.gov/gap/) data with accession of phs000452.v3.p1 [19]. We obtained normalised expression data and clinical data from the TIGER web portal (http://tiger.canceromics.org/#/home).

### 2.2 Single‐Cell RNA Sequencing (scRNA‐Seq) Data Collection and Pre‐Processing

ScRNA‐seq of CRC patients in Lee et al.'s study was obtained from the GEO database (GSE132465) [20]. We extracted 10 CRC samples and 10 matched normal mucosa samples for analysis. For tumour samples and normal samples, respectively, R packages ‘Seurat’ were used to create a Seurat object from scRNA‐seq data [21]. Cells with > 1000 unique molecular identifier (UMI) counts; > 200 genes and < 6000 genes; and < 20% of mitochondrial gene expression in UMI counts were reserved. A total of 22,793 cells from CRC samples and 16,404 cells from normal samples were selected. Followed by ‘LogNormalize’ cell normalisation and scaling, the top 1000 highly variable genes were selected for principal component analysis (PCA). Then, the first 30 PCs were selected for clustering with a resolution of 0.6. Cells were visualised using a two‐dimensional t‐distributed stochastic neighbour embedding (t‐SNE) on the same distance metric. The ‘FindAllMarkers’ function was used to screen the marker genes with logfc.threshold = 0.25, min.pct = 0.1 and *p*_val_adj = 0.05. Cell‐type annotation was performed with ‘SingleR’ package using Human Primary Cell Atlas Data provided by ‘celldex’ package [22].

### 2.3 Identification of T‐Cell Sub‐Populations

We extracted T cells annotated by SingleR and identified sub‐populations. The classic workflow in Seurat was used to perform dimension reduction and unsupervised clustering with the first 20 PCs and a resolution of 0.4 for T cells in tumour samples and normal samples respectively. Top 10 differentially expressed genes (DEGs) were identified as markers in clusters. Cell types were manually annotated according to the cell markers recorded in CellMarker 2.0 (http://117.50.127.228/CellMarker/) and ScType (http://session.asuscomm.com/) database. The criteria for cell‐type annotation are as follows: (1) First, markers of ‘Colon’ and ‘Colorectum’ tissue in CellMark2.0 were used for cluster annotation. (2) Besides, when there is no accurate label for annotation, we comprehensively considered markers in the label of ‘immune system’ both in CellMark2.0 and ScType. (3) Then, the immune markers in other solid tissues, in which histological locations are close to the colorectal, were used for annotation. The markers of exhausted CD8+ T cells were obtained from CellMark2.0.

### 2.4 Trajectory Inference and Pseudo‐Time Ordering for T Cells

Single‐cell pseudo‐time trajectories were constructed for T cells with R packages ‘monocle3’ [23]. Raw expression matrix of T cells was extracted and normalised using Monocle's size factor normalisation method. The uniform manifold approximation and projection (UMAP) algorithm was employed to project PCA of the cells into two‐dimensional space. The principal graph was learned from the UMAP space using the ‘learn_graph’ function, using the predefined clusters as guidance. The pseudo‐time value for each cell was computed based on the principal graph, using the ‘order_cells’ function. We set the node that has the most branches as the root of the principal graph.

### 2.5 Analysis of Cell–Cell Communication

The cell–cell communications among cell types were analysed using the R package ‘CellChat’ [24]. We focused on the human database in CellChat and identified over‐expressed ligands or receptors by ‘identify Over Expressed Genes’ and ‘identify Over Expressed Interactions’ functions. Then, we mapped gene expression data onto protein–protein interaction network. The ‘compute Commun Prob’ and ‘filter Communication’ functions were used to compute communication probability and infer cellular communication network. The ‘compute Commun Prob Pathway’ and ‘aggregate Net’ functions were used to infer the cell–cell communication at a signalling pathway level between each cell type.

### 2.6 Identification of Prognosis‐Related Genes and Construction of Prognostic Model

The distributed gradient‐boosted decision tree machine learning algorithm Extreme Gradient Boosting (XGBoost) was used to identify clinical outcome‐related genes and survival time‐related genes. We identified 197 and 274 genes associated with outcome and survival time by R package ‘xgboost’ in TCGA COAD samples. Finally, a total of 34 genes were overlapping in the top 100 outcome‐related genes and top 100 survival time‐related genes.

Multivariate Cox regression models were constructed based on optimised genes by R package ‘survival’. Risk score was calculated by taking into account the expression of 34 genes and coefficients in the multivariate Cox regression model.

The risk score was calculated as follows:

$$\text{Risk score}=\Sigma \left({\text{Expi}}^{*}\;\text{coefi}\right)$$

### 2.7 Survival Analysis

We classify patients based on the median risk score. The log‐rank test was used to evaluate the difference in survival time between high‐risk scoring patients and low‐risk scoring patients, using the R package ‘survival’.

### 2.8 Evaluation of Immune Infiltration

Cell‐type identification by estimating relative subsets of RNA transcripts (CIBERSORT) method was used to accurately estimate the relative proportions of various cell subsets of COAD and READ samples in TCGA [25]. The ‘ComplexHeatmap’ and ‘ggplot2’ R package were used to visualise the abundance of 22 immune cells of all samples. The differences in immune checkpoints (CD274, CTLA4, CD47 and BTLA) expression between high‐ and low‐risk groups were analysed using one‐sided Wilcoxon rank‐sum test.

### 2.9 Cell Culture

The SW480, HCT116, Lovo and RKO cell lines derived from human colorectal cancer (CRC), along with the NCM460 cell line derived from human colon epithelium, were obtained from Servicebio (Wuhan, China). The cells were cultured in DMEM supplemented with 10% foetal bovine serum (FBS) obtained from Servicebio (Wuhan, China). Incubation of all cell types took place in a humidified environment with 5% CO_2_ at a temperature of 37°C. The cultural environment required the cell medium to be changed every 2 days.

### 2.10 Quantitative Real‐Time Polymerase Chain Reaction (qRT‐PCR)

The extraction of total RNA was performed using a DNA Mini‐prep kit (Sangon Biotech Co. Ltd.) in cells. After assessing the concentration and quality of RNA, reverse transcription was performed using PrimerScript RT Master mix provided by Takara Biotechnology Co. Ltd. All PCR primers were supplied by Sangon Biotechnology. The SYBR Green Master Mix (Thermos Fisher Scientific) was employed for conducting the quantitative PCR, utilising the ABI 9300 PCR system from Applied Biosystems. The relative expression levels were normalised against GAPDH as an internal reference and calculated using the formula 2^−△△^CT.

### 2.11 Cell Viability

The cell viability of CRC cells was assessed using a cell counting kit‐8 (CCK‐8) obtained from Servicebio in Wuhan, China. The CRC cells were cultured in 96‐well plates for different time periods (24, 48 and 72 h). Subsequently, each well was supplemented with 90 μL of DMEM and 10 μL of CCK‐8 working solution. The cells were incubated at a temperature of 37°C for 1 h and subsequently measured at a wavelength of 450 nm using a SpectraMax spectrophotometer (Molecular Devices) to determine the optical density (OD) values.

### 2.12 Transwell Assay

To perform the Transwell assay, a suspension of 5 × 104 cells/well in 200 μL serum‐free medium was added to the upper chamber. In contrast, the lower chamber was filled with 600 μL medium containing 10% FBS (8‐μm pore size, Costar, Corning, USA). After a 24‐h incubation period (invasion assay) in a humidified environment with 5% CO_2_ at 37°C, the cells within the parietal chamber were extracted. Subsequently, the cells on the submucosal surface were treated with 4% paraformaldehyde for 30 min and subjected to staining using a crystal violet solution. The migrated cells were subsequently quantified under a microscope in four randomly selected regions of each membrane layer.

### 2.13 Statistical Analysis

The difference of infiltration immune cell type between tumour and normal samples was analysed using one‐sided Wilcoxon rank‐sum test. One‐sided Wilcoxon rank‐sum test was used to test the difference of risk score between clinical stages or treatment response groups. All statistical analyses were performed using R software, and *p* < 0.05 was considered statistically significant.

## 3 Results

### 3.1 Resolution of Immune Microenvironment in Colorectal Cancer

**FIGURE 1:**
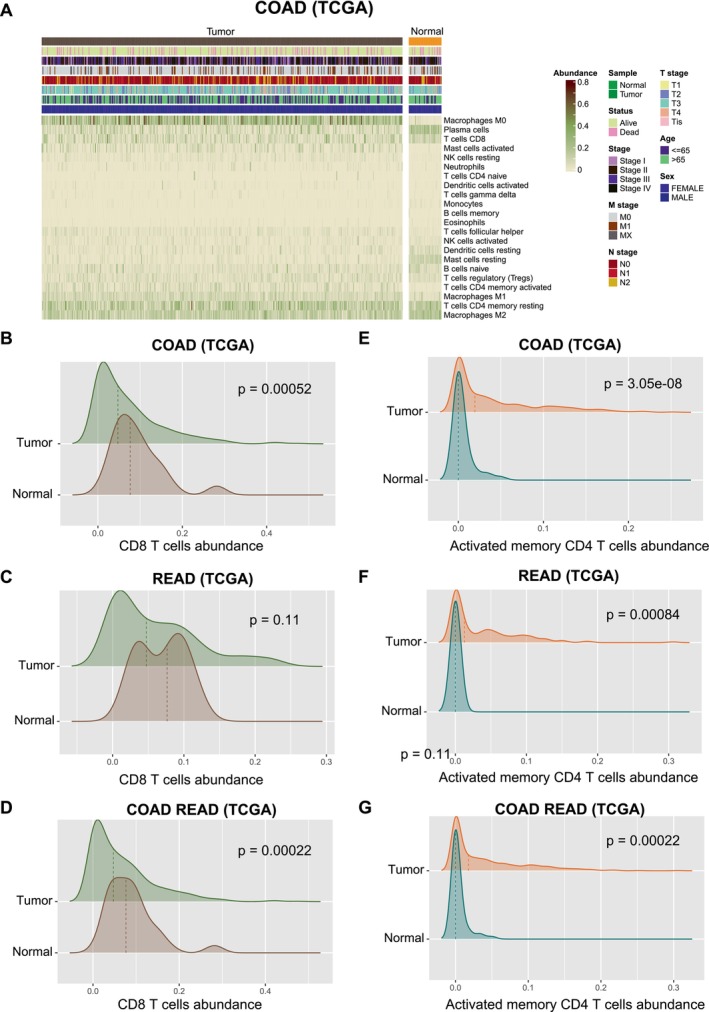
Differential infiltration of T cells between colorectal cancer and normal samples. (A) The heatmap shows proportion of 22 immune cell infiltration between COAD and matched normal samples in TCGA. The top stripes mean clinical information of corresponding patients. (B–D) Differential infiltration of CD8 T cells between COAD, READ or colorectal cancer (COAD and READ) samples and matched normal samples. (E–G) Differential infiltration of activated memory CD4 T cells between COAD, READ or colorectal cancer (COAD and READ) samples and matched normal samples.

The infiltration of immune cells is associated with tumour malignancy and treatment. Our study explored immune infiltration in colorectal cancer and therapeutic relevance. The analytical process in this study is illustrated in Figure S1. To characterise the immune microenvironment of COAD, CIBERSORT was performed to estimate the proportion of 22 immune cells infiltration in bulk TCGA samples (Figure 1A). In colorectal cancer samples, the infiltration of CD8 T cells was lower than that in normal samples [Figure 1B–D, one‐sided Wilcoxon rank‐sum test, COAD (TCGA), CD8 T cells *p* = 0.00052; READ (TCGA), CD8 T‐cells abundance *p* = 0.11; COAD READ (TCGA), CD8 T‐cells abundance *p* = 0.00022]. The infiltration of activated memory CD4 T cells was significantly higher in colorectal cancer samples than in normal samples [Figure 1E–G, *p* < 0.05, one‐sided Wilcoxon rank‐sum test, COAD (TCGA), activated memory CD4 T‐cells abundance *p* = 3.05e‐08; READ (TCGA), activated memory CD4 T‐cells abundance *p* = 0.00084; COAD READ (TCGA), activated memory CD4 T‐cells abundance *p* = 0.00022].

### 3.2 Differential Infiltration of T Cell Between Colorectal Cancer and Normal Samples

**FIGURE 2:**
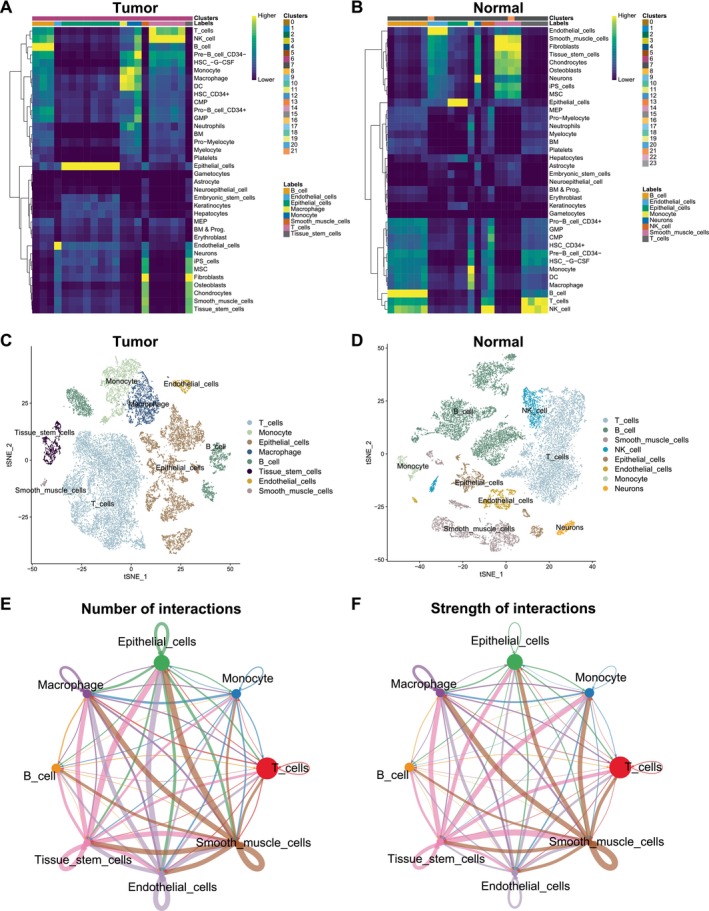
Resolution of immune microenvironment in colorectal cancer and matched normal samples by scRNA‐seq. (A,B) The SingleR method was used for cell annotation in colorectal cancer and matched normal samples. The heatmaps show the assignment score for each cell and label. (C,D) The tSNE plots show the annotations of cell types by SingleR method in colorectal cancer and matched normal samples. (E,F) Numbers and strength of interactions among cell types in colorectal cancer single cells.

To further investigate immune infiltration of cell types between colorectal cancer and normal samples, we obtained scRNA‐seq data for 10 samples (GSE132465) from GEO. The gene expression matrix was pre‐processed by Seurat workflow. We identified DEGs in each cluster by the ‘Find All Markers’ function, the top five significant DEGs of clusters were visualised by heatmap (Figure S2). By SingleR method, the cell types in colorectal cancer and normal samples were annotated (Figure 2A,B). There were eight major cell types in tumour samples, including T cells, monocytes, epithelial cells, macrophages, B cells, tissue stem cells, endothelial cells and smooth muscle cells (Figure 2C). There were also eight cell types in normal samples, but without tissue stem cells and macrophages existing, in addition, neurons and NK cells were newly increasing (Figure 2D). The proportion of T cells in tumour samples was higher than normal samples, but there was not a great disparity of the distinction. To explore the mutual effects in tumour microenvironment, we next performed cell–cell communication analysis by Cell Chat. Numbers and strength of interactions among cell types in tumour samples are shown in Figure 2E,F. Epithelial cells, tissue stem cells and smooth muscle cells had frequent communications. T cells have more communications with epithelial cells than B cells, less than macrophages.

### 3.3 Definition of T‐Cell Sub‐Populations

**FIGURE 3:**
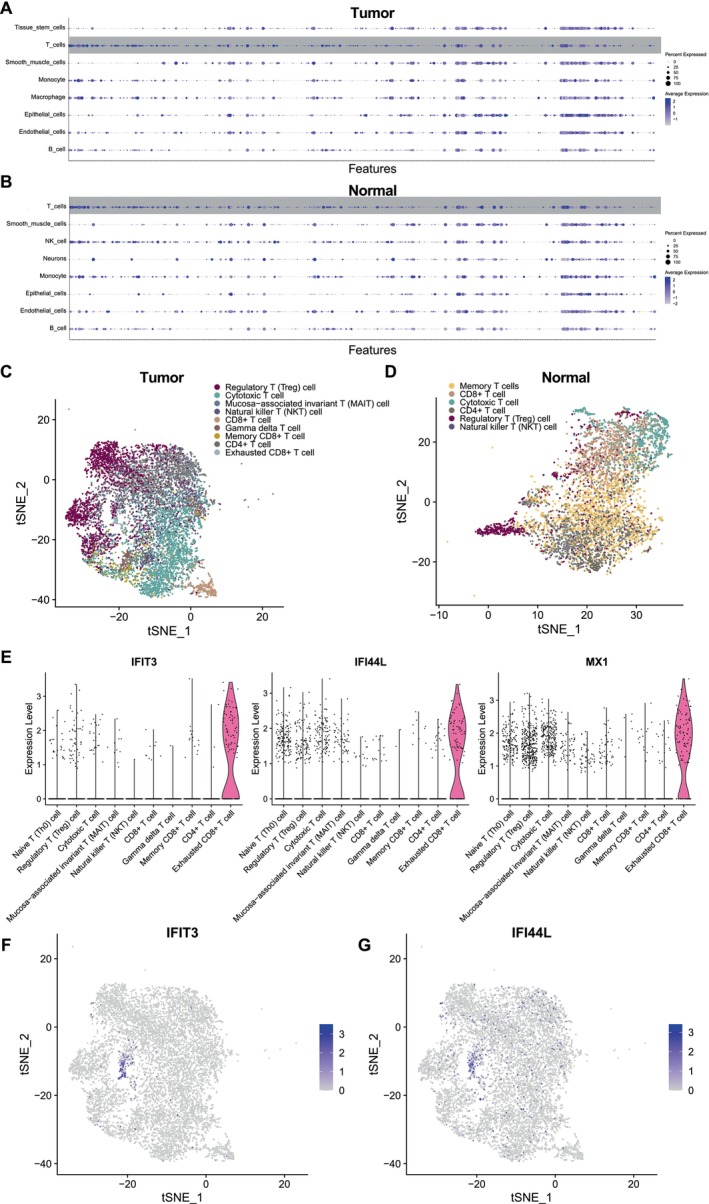
Identification of T‐cell sub‐populations. (A,B) The expression of cell markers of T cells which provided in SingleR in tumour and normal samples. (C,D) The tSNE plots showed the annotations of T‐cell sub‐populations in tumour and normal samples. (E) The expression of three exhausted CD8+ T‐cell markers (IFIT3, IFI44L and MX1) which are provided by CellMarker2.0 among T‐cell sub‐populations. (F,G) The tSNE plots are coloured by the expression of IFIT3 and IFI44L.

To further explore the function of T cells, we re‐analysed single‐cell transcriptomes of T cells and identified cell sub‐populations (Figure S3). The expression of T‐cell markers that were provided in SingleR of tumour and normal samples is shown in Figure 3A,B. According to a manual annotation that referred to specialised databases, we identified nine T‐cell sub‐populations [regulatory T (Treg) cell, cytotoxic T cell, mucosa‐associated invariant T (MAIT) cell, natural killer T (NKT) cell, CD8+ T cell, gamma–delta T cell, memory CD8+ T cell and exhausted CD8+ T cell]in tumour sample (Figure 3C). Besides, there were six T‐cell sub‐populations [memory T cell, CD8+ T cell, cytotoxic T cell, CD4+ T cell, regulatory T (Treg) cell and natural killer T (NKT) cell] identified in normal samples (Figure 3D). We found that the exhausted CD8+ T cells were exclusive to tumour samples. Figure 3E–G shows the expression of three exhausted CD8+ T‐cell markers (IFIT3, IFI44L and MX1) that were provided by CellMarker2.0 among T‐cell sub‐populations.

**FIGURE 4:**
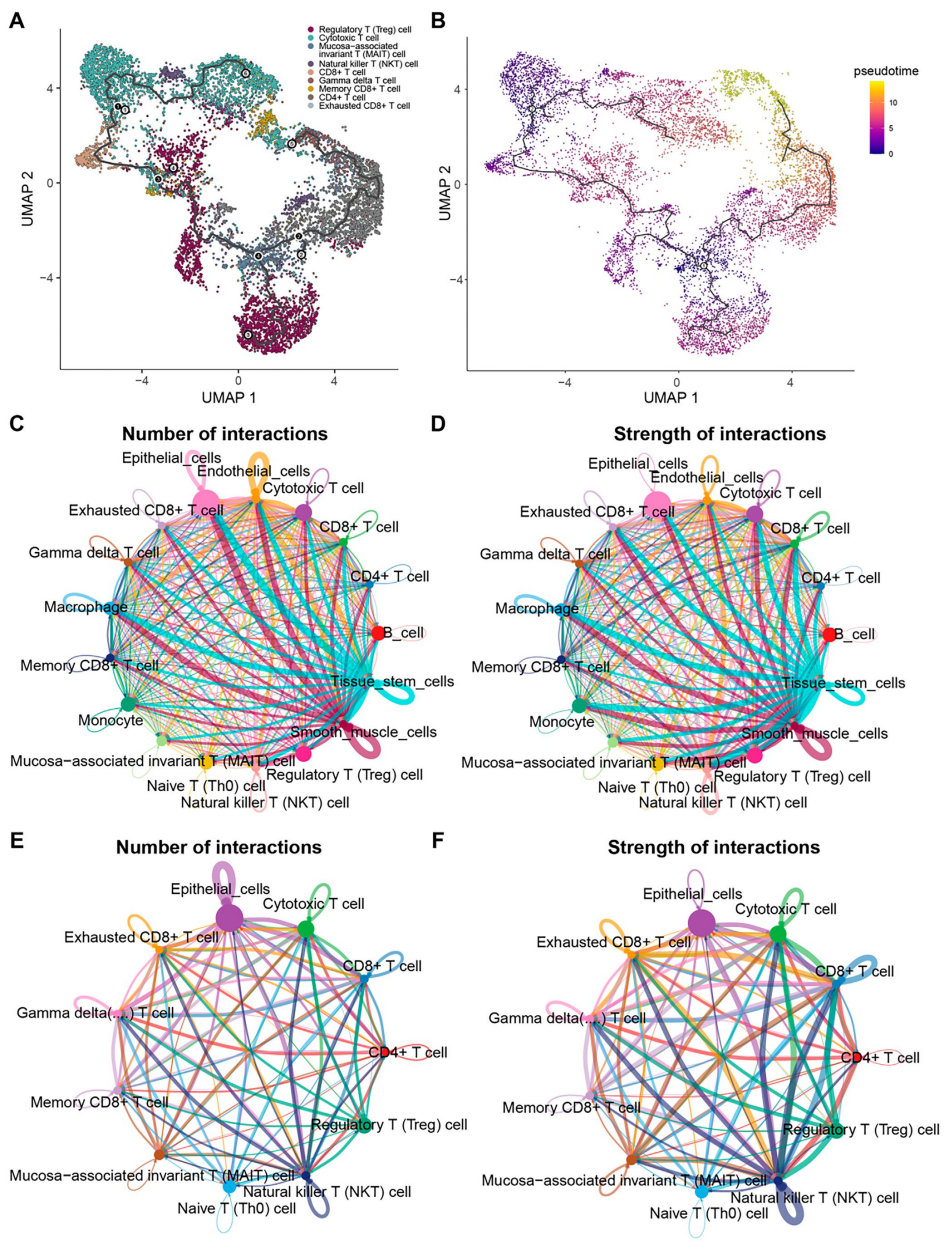
Analysis of pseudo‐time trajectory and cell–cell communications. (A) The UMAP plot shows the identification of T‐cell sub‐populations trajectory by monocle3, the points are coloured by T‐cell sub‐population. (B) The UMAP plot shows the pseudo‐time value. (C,D) Numbers and strength of interactions among all cell types in tumour samples. (E,F) Numbers and strength of interactions among epithelial cells and T‐cell sub‐populations in tumour samples.

To explore the developmental trajectories of T‐cell sub‐populations, we performed pseudo‐temporal ordering of the T cell using Monocle3 (Figure 4A). We set two nodes that have the most branches as the root of the principal graph; exhausted CD8+ T cells appears at the end of branches in pseudo‐temporal routes (Figure 4B).

**FIGURE 5:**
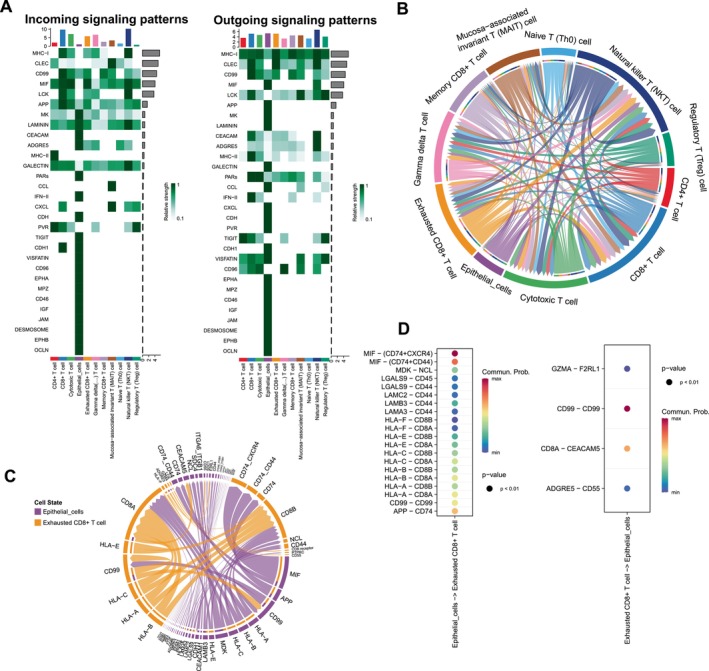
Analysis of cell communications between exhausted CD8+ T cells and epithelial cells. (A) Heatmap visualising the possible incoming or outgoing signalling pathways among T‐cell sub‐populations and epithelial cells. (B) Chord diagram shows interactive pathways among T‐cell sub‐populations and epithelial cells. (C) Chord diagram indicates interactive signalling pairs between epithelial cells and exhausted CD8+ T cells. (D) Dot plot visualising the possible incoming or outgoing signalling pairs between epithelial cells and exhausted CD8+ T cells.

To further elucidate the role of all cell types, we performed cell–cell communication analysis in tumour samples (Figure 4C,D). Epithelial cells frequently communicated with other cells, including immune cells. We next explored the communications between epithelial cells and all T‐cell sub‐populations (Figure 4E,F). Epithelial cells have frequent communications with exhausted CD8+ T cells. Then, we investigated the potential outgoing and incoming signals among epithelial cells and T‐cell sub‐populations (Figure 5A). The epithelial cells received a lot of communications from MK, LAMININ, CEACAM and ADGRE5 pathways. The exhausted CD8+ T cells received more communications about the MIF pathway and sent more signals of MHC‐I and CLEC pathways. Figure 5B shows the interactive pathways among epithelial cells and all T‐cell sub‐populations. Subsequently, the specific signal pairs between epithelial cells and exhausted CD8+ T cells were investigated (Figure 5C,D). The results showed strong communication between exhausted CD8+ T cells through the MIF‐(CD74 + CXCR4), MIF‐(CD74 + CD44) and CD99‐CD99.

### 3.4 Construction of the Prognostic Model

**FIGURE 6:**
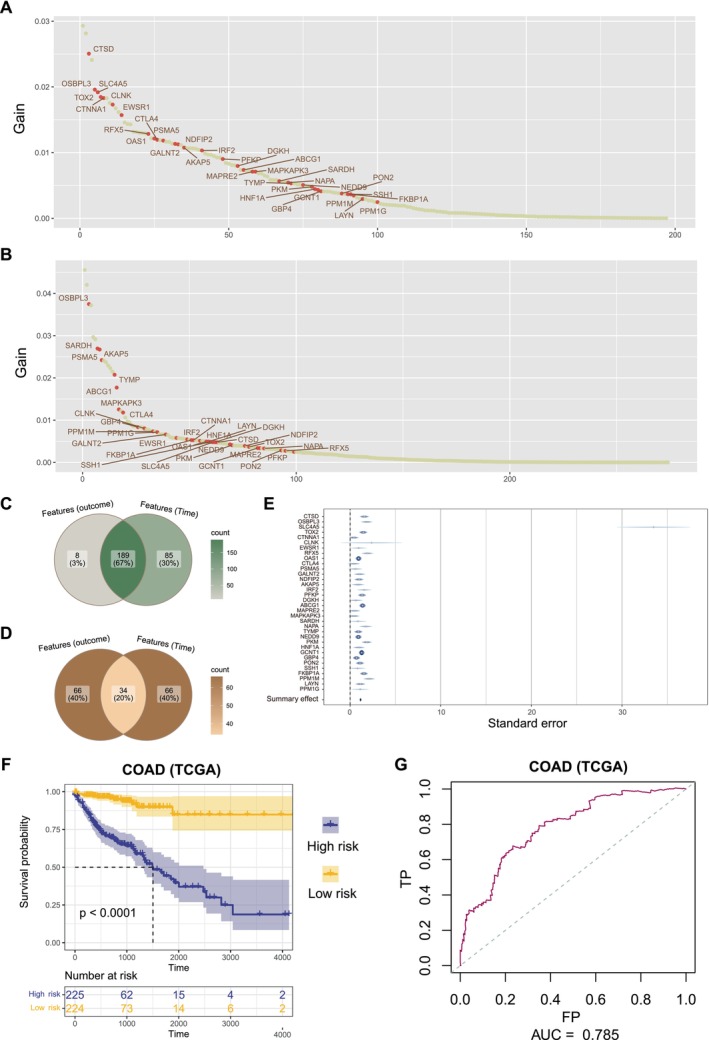
Construction and validation of the prognostic model. (A,B) Distribution of gain value for patient outcome‐related genes and survival time‐related genes identified by XGBoost machine learning algorithm. The red points means 34 final determined genes. (C) The overlapping of all outcome‐related genes and survival time‐related genes. (D) The overlapping of top 100 outcome‐related genes and top 100 survival time‐related genes. (E) The multivariate Cox proportional hazard model was built based on 34 genes. (F) Log‐rank test was used to assess the difference in OS between high‐risk and low‐risk samples in TCGA COAD cohort. (G) ROC curve of prognostic model for 3 years prediction in TCGA COAD cohort.

We next explored the prognostic effect of exhausted CD8+ T cells in COAD. We first collected markers of exhausted CD8+ T cells that were provided in CellMarker2.0, along with the top 10 DEGs of the exhausted CD8+ T‐cell cluster in tumour samples. By XGBoost machine learning, we identified 197 outcome‐related markers in the TCGA COAD cohort (Figure 6A) and identified 274 survival time‐related markers (Figure 6B). There were 189 overlapping genes (Figure 6C). Then, we selected 34 genes that were overlapping in the top 100 outcome‐related genes and the top 100 survival time‐related genes to construct a multivariate Cox regression model (Figure 6D). Figure 6E shows the standard error of the 34 genes in the multivariate Cox regression model.

According to the prognostic model, we calculated the risk score of each patient in the TCGA COAD cohort. Patients were classified into the high‐risk and low‐risk groups using the median as the cutoff value (Figure S4). Patients in the high‐risk group had a significantly poorer overall survival (OS) (Figure 6F, *p* < 0.0001, log‐rank test). According to the area under the curve (AUC) of the receiver operating characteristic (ROC) curve, the risk score was able to predict 3‐year survival for patients (Figure 6G, AUC = 0.79).

### 3.5 Evaluation of the Predictive Efficiency

**FIGURE 7:**
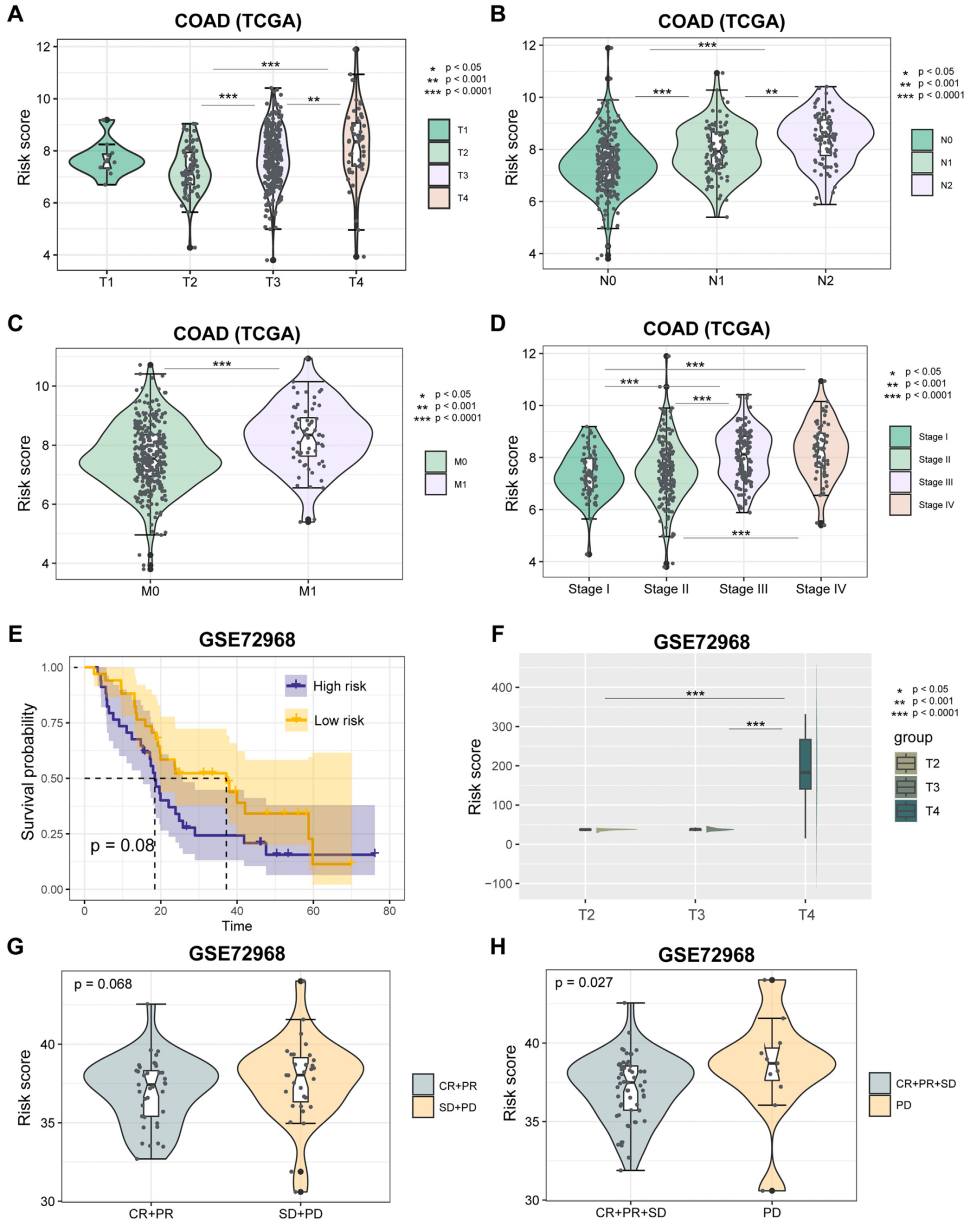
Prognosis power of tumour stages and treatment. (A–D) Distribution of risk score among T groups, N groups, M groups or tumour stage groups. (E) Log‐rank test was used to assess the difference in OS between high‐risk and low‐risk samples in COAD cohort (GSE72968). (F) Distribution of risk score among T groups in GSE72968. (G) Distribution of risk score between chemotherapy response (CR and PR) and non‐response (SD and PD) group. (H) Distribution of risk score between chemotherapy response (CR, PR and SD) and non‐response (PD) group.

We further examined the distribution of risk scores across different clinical tumour stage groups. The risk score in T3 and T4 stages was significantly higher than T2 in the TCGA COAD cohort (Figure 7A, *p* < 0.001, one‐sided Wilcoxon rank‐sum test). The risk score in N2 and N3 stages was significantly higher than N1 in the TCGA COAD cohort (Figure 7B, *p* < 0.001, one‐sided Wilcoxon rank‐sum test). The risk score in the M1 stage was significantly higher than M0 in the TCGA COAD cohort (Figure 7C, *p* < 0.001, one‐sided Wilcoxon rank‐sum test). The risk score in stages III and IV was significantly higher than in stages I and II in the TCGA COAD cohort (Figure 7D, *p* < 0.0001, one‐sided Wilcoxon rank‐sum test).

Subsequently, we evaluated the predictive efficacy of the risk score in independent validation datasets. Using the multivariate Cox proportional hazard model, we calculated the risk scores for patients in the GSE72968 dataset. Patients were stratified into high‐risk and low‐risk groups based on the median risk score. Those in the high‐risk group exhibited a trend towards poorer overall survival (Figure 7E, *p* = 0.08, log‐rank test). The risk score in T4 stage was significantly higher than T2 and T3 in the GSE72968 cohort (Figure 7F, *p* < 0.0001, one‐sided Wilcoxon rank‐sum test). In addition, we analysed the predictive effect on chemotherapy response. The risk score of non‐responsive [SD (stable disease) and PD (progressive disease) or only PD] patients was significantly higher than responsive patients [CR (complete response) and PR (partial response), or CR, PR and SD] (Figure 7G,H; *p* = 0.078, *p* = 0.027, one‐sided Wilcoxon rank‐sum test).

### 3.6 Prediction of Immunotherapy Response

**FIGURE 8:**
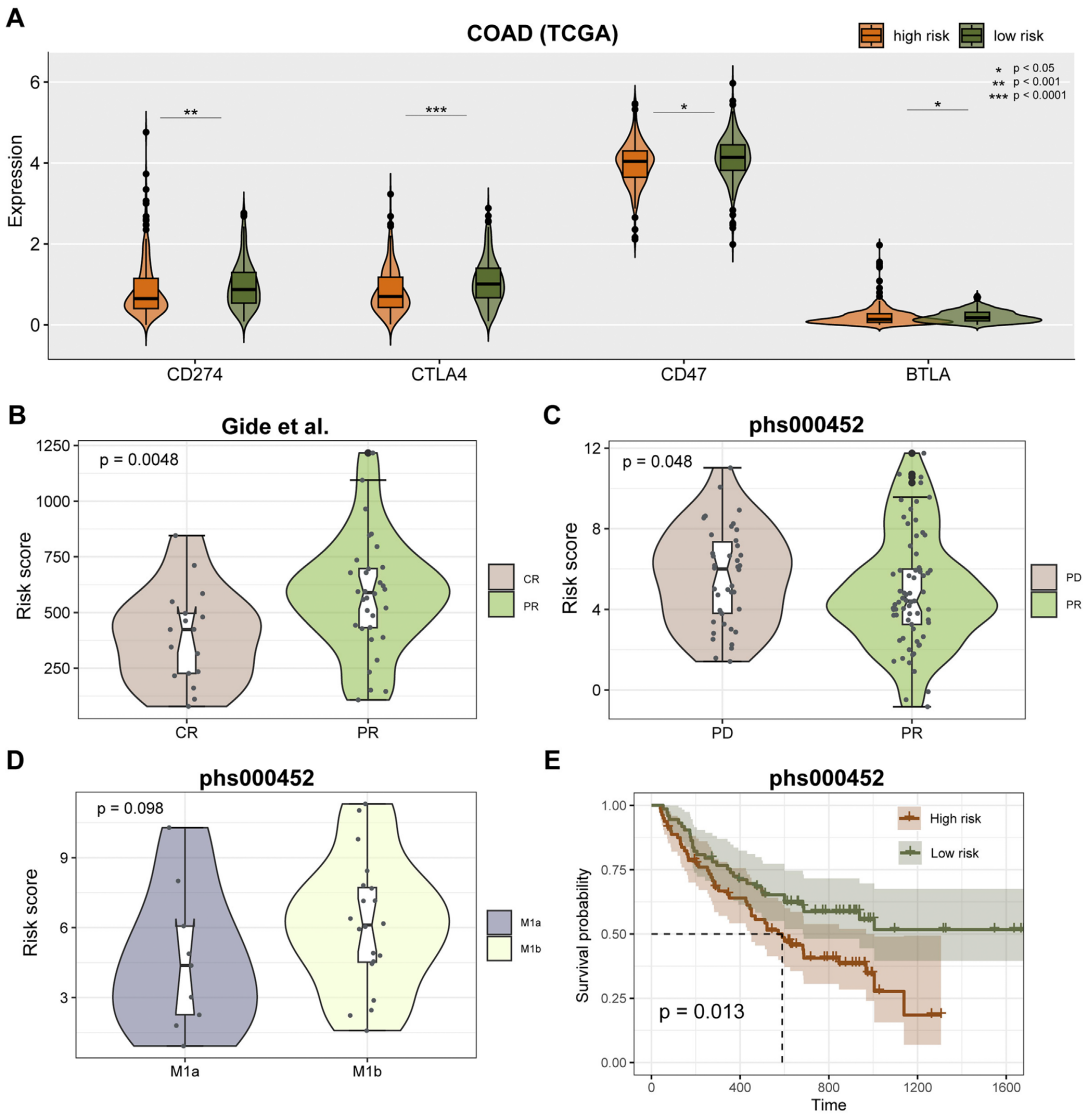
Prognosis power of model to immunotherapy. (A) Expression of CD274, CTLA4, CD47 and BTLA between high‐risk and low‐risk score groups in TCGA cohort. (B) Distribution of risk score between immunotherapy CR and PR group in Gide et al. dataset. (C) Distribution of risk score between immunotherapy PD and PR group in phs000452 dataset. (D) Distribution of risk score between M1a and M1b stage in phs000452 dataset. (E) Survival analysis for phs000452 samples by log‐rank test.

The different immune microenvironments in tumours are closely related to the response to immunotherapy. Here, we did an analysis of the correlation between the risk score and the immunotherapy response. The expression of immune checkpoints CD274 (PD‐1), CTLA4, CD47 and BTLA in the high‐risk group was significantly higher than in the low‐risk group in the TCGA COAD cohort (Figure 8A, *p* < 0.05, one‐sided Wilcoxon rank‐sum test). Next, we explored if the risk model can predict the response to immunotherapy. The risk score in patients with PR was significantly higher than in patients with CR in the Gide et al. dataset (Figure 8B, *p* = 0.0048, one‐sided Wilcoxon rank‐sum test). Besides, the risk score in patients with PD was significantly higher than in patients with PR in the phs000452 dataset (Figure 8C, *p* = 0.0048, one‐sided Wilcoxon rank‐sum test). The risk score in the M1b stage was higher than in the M1a stage in the phs000452 dataset (Figure 8D, *p* = 0.098, one‐sided Wilcoxon rank‐sum test). In addition, the survival analysis showed that patients in the high‐risk group had a significantly poorer OS in the phs000452 dataset (Figure 8E, *p* = 0.013, log‐rank test).

At the same time, we assessed the relative mRNA expression of MIF in human colorectal cancer cell lines SW480, HCT116, Lovo and RKO as well as in normal colon epithelial cells NCM460 by referring to Figure S5A. The levels of MIF were higher in Lovo and HCT116 cells, moderate in SW480 and RKO cells and lower in NCM460 cells. Afterwards, we generated HCT116 cells with MIF knock‐down (Figure S5B). Successful confirmation of the modulation of MIF expression level in HCT116 cells was achieved through qRT‐PCR. The results of the CCK‐8 assay demonstrated that MIF exerted a stimulatory effect on the proliferation of CRC cells, as depicted in Figure S5C. Moreover, in vitro analysis revealed that inhibition of MIF led to a reduction in invasion by Lovo and HCT116 cells (Figure S5D). Collectively, these findings suggest that MIF functions as a growth and infiltration promoter for CRC cells.

## 4 Discussion

In the 1970s, researchers first realised that T cells could recognise and kill target cells through MHC molecules [26]. With the widespread use of immunotherapy, the role of CD8+ T cells has been intensively explored [27, 28, 29]. Currently, research on CD8+ T‐cell exhaustion has focused on two main areas: the search for molecular markers of exhaustion and the investigation of the mechanisms of exhaustion [30, 31, 32]. In the context of searching for molecular markers of exhaustion, researchers have identified several molecules associated with CD8+ T‐cell exhaustion, such as PD‐1, Tim‐3 and LAG‐3 [33, 34, 35]. Such molecules can be used as diagnostic markers for CD8+ T‐cell exhaustion and also as therapeutic targets. In terms of mechanism, researchers believe that factors such as suppressive immune microenvironment and inflammation contribute to the dysfunction and metabolism of CD8+ T cells [36, 37, 38], while the mechanism of intercellular communication that causes CD8+ T‐cell exhaustion and its impact on the prognosis of immunotherapy remains to be explored in depth.

In this study, we analysed sequencing data of CRC tumour samples from the TCGA database and found that the abundance of CD8+ T‐cell infiltration in tumour tissue was significantly reduced in CRC patients compared to paired normal tissue. This phenomenon may be due to the presence of an immunosuppressive microenvironment. Cytokines such as IL‐35 have been reported to inhibit the activity of CD8+ T cells in the tumour microenvironment by suppressing their proliferation and cytokine secretion, leading to a decrease in infiltration abundance [39]. In addition, studies have shown that a variety of chemicals released from tumour cells, such as PGE2, can inhibit the chemotaxis, proliferation and function of CD8+ T cells [40]. Also, the apoptosis of CD8+ T cells in tumour tissues contributes to their reduced infiltration abundance. Exosomes (Exo) released from tumour cells can induce apoptosis in CD8+ T cells by inducing them to express death receptors such as FasL and TRAIL [41]. In addition, tumour cells can release a variety of cytokines and chemicals such as IL‐10, TGF‐β, PGE2 and IDO to induce apoptosis in CD8+ T cells, and these cytokines and chemicals may induce apoptosis in CD8+ T cells through a variety of signalling pathways and routes [36].

Then, we obtained scRNA‐seq sequencing data for colon cancer and its normal controls by accessing the GEO database (GSE132465). Based on these data, we compared and analysed the immune cell infiltration in tumour tissues and normal tissues. Our results showed the presence of tissue‐specific stem cells and macrophages in tumour tissue compared to normal mucosal tissue, as well as a lack of neuronal and NK cell infiltration. Interestingly, the number of infiltrating T cells in tumour tissue was instead slightly higher than in normal tissue, suggesting that quantity is not the only determinant, but rather that the functional status of T cells is altered. Therefore, we believe that a more in‐depth study of T‐cell subtypes in tumour cells is warranted.

We employed the SingleR method to identify nine T‐cell sub‐populations in the tumour samples. By pseudo‐temporal trajectory analysis, we found that some sub‐populations of CD8+ T cells appeared at the end of pseudo‐temporal pathways, suggesting that these CD8+ T cells had reached the end of their differentiation and were unable to develop further into other subtypes of cells. Using the combined results of intercellular communication and pathway analysis, we found a strong connection between depleted CD8+ T cells and epithelial cells. Communication between these two cell types occurs mainly through the MIF‐(CD74 + CXCR4), MIF‐(CD74 + CD44) and CD99‐CD99 pathways. This suggests that MIF and CD99 may induce CD8+ T‐cell exhaustion. Chen et al. reported that blocking MIF reduces the progression of vitiligo by inhibiting the activation and growth of CD8+ T cells [42]. Winger et al. found CD99 represents a promising therapeutic target for controlling T‐cell‐driven autoimmune diseases affecting the central nervous system [43]. TF is thought to act mainly by enhancing cell‐mediated immunity, which results in the production of migration inhibitory factor (MIF) and interferon gamma (IFN‐γ) [44]. In our data, we knocked down the expression of MIF in CRC cells. We found knock‐down MIF can inhibit cell growth, migration as well as induce apoptosis. MIF (macrophage migration inhibitory factor) is an immunomodulatory factor. Cui et al. showed that MIF increases CD8+ T‐cell expression of PD‐1 and Tim‐3 through activation of NF‐κB and PI3K/AKT signalling pathways [45], thereby promoting CD8+ T‐cell exhaustion. Wang et al. showed that MIF also regulated the expression of the transcription factor HIF‐1α, which increased the expression of PD‐1, Tim‐3 and LAG‐3 in CD8+ T cells, leading to impaired function and degradation of CD8+ T cells [46]. Zhang et al. showed that MIF promoted the expression of PD‐1 and Tim‐3 in CD8+ T cells by regulating the levels of ROS [47]. CD99 is a transmembrane protein, also known as MIC2, which was originally identified in human leukaemia cells and is thought to be a marker for the recognition of tumour cells. CD99 expression levels were significantly associated with impaired function and PD‐1 expression in CD8+ T cells from human liver cancer patients. To the best of our knowledge, CD8+ T cells deficient in PD‐1 and LAG‐3 exhibit enhanced anti‐tumour immunity through autocrine, cell‐intrinsic IFN‐γ signalling [48]. By engaging carcinoembryonic antigen‐related cell adhesion molecule 1 (CEACAM‐1), soluble Tim‐3 (sTim‐3) induces terminal T‐cell exhaustion and impairs the efficacy of CD8+ T‐cell responses to PD‐1 blockade [49]. Upon T‐cell activation, IRF4 up‐regulation modulates PD‐1 expression, reduces IFN‐γ production and diminishes the proliferative capacity of tumour‐infiltrating lymphocytes (TILs). This study also showed that by inhibiting CD99 expression, CD8+ T‐cell function could be restored and drug resistance in patients with hepatocellular carcinoma could be reduced. CD99 may promote CD8+ T‐cell depletion by regulating PD‐1 expression. A study by Chen et al. showed that CD99 regulates the metabolic pathway of CD8+ T cells by interacting with PDK1 [50]. CD99 deficiency promotes the metabolic activity and function of CD8+ T cells and enhances the efficacy of immunotherapy. This suggests that CD99 may influence the function and depletion status of CD8+ T cells by regulating metabolic pathways.

We successfully identified 34 markers that correlate strongly with CD8+ T‐cell exhaustion through data analysis and screening of public databases. Using these markers, we constructed patient prognosis prediction models and immunotherapy responsiveness models using XGBoost machine learning algorithms. Through a comprehensive analysis of a cohort of patients receiving immunotherapy, we demonstrate that these models have high accuracy in predicting survival in colorectal cancer. We observed that in the TCGA COAD cohort, risk scores were significantly higher in patients with stages T3 and T4 than in T2, and similar results were obtained in the validation cohort. In addition, we further explored whether risk models could predict response to immunotherapy. In the Gide et al. dataset, risk scores were significantly higher in PR patients than in CR patients. Survival analysis showed that patients in the high‐risk group had significantly lower overall survival in the phs000452 dataset. In summary, the risk score model has a promising accuracy for predicting response to immunotherapy and patient prognosis and can be taken as a good predictor of immunotherapy and chemotherapy. At the same time, we tested the cell growth and invasion of MIF suppression in CRC cells. Our data show that suppression of MIF in CRC cells can inhibit both cell proliferation and invasion.

In summary, this study investigated the mechanism of CD8+ T‐cell exhaustion generation in CRC tumour tissues from the perspectives of intercellular communication and cell differentiation. MIF and CD99 were potentially valuable regulators for inducing an exhaustion phenotype in CD8+ T cells. In addition, this study constructed a prognostic and immunotherapeutic responsiveness prediction model based on CD8+ T‐cell depletion markers. The validation results of the external validation set showed that our model has good predictive efficiency.

## Author Contributions

**Xiao‐Hua Ling:** conceptualization (equal), data curation (equal), formal analysis (equal), funding acquisition (equal), investigation (equal), methodology (equal), project administration (equal), resources (equal), software (equal), supervision (equal), validation (equal), visualization (equal), writing – original draft (equal). **Gang Chen:** conceptualization (supporting), data curation (supporting), formal analysis (supporting), funding acquisition (supporting), investigation (equal), methodology (supporting), project administration (supporting), resources (supporting), software (supporting), supervision (supporting), validation (supporting), visualization (supporting), writing – original draft (equal). **Nan‐Nan Liu:** conceptualization (supporting), data curation (supporting), formal analysis (supporting), funding acquisition (supporting), investigation (supporting), methodology (supporting), project administration (supporting), resources (supporting), software (supporting), supervision (supporting), validation (supporting), visualization (supporting), writing – original draft (supporting). **Wen‐Xin Xu:** conceptualization (supporting), data curation (supporting), formal analysis (supporting), funding acquisition (supporting), investigation (supporting), methodology (supporting), project administration (supporting), resources (supporting), software (supporting), supervision (supporting), validation (supporting), visualization (supporting), writing – original draft (supporting). **Ming‐Feng Ding:** conceptualization (lead), data curation (equal), formal analysis (equal), funding acquisition (lead), investigation (equal), methodology (supporting), project administration (supporting), resources (equal), software (equal), supervision (equal), validation (equal), visualization (equal), writing – original draft (equal).

## Disclosure

The authors declare no conflicts of interest.

## Ethics Statement

The animal studies were approved by the Institutional Animal Care and Use Committee at Fourth Harbin Medical University (Cat. 20230215100642).

## Consent

All claims expressed in this article are solely those of the authors and do not necessarily represent those of their affiliated organisations or those of the publisher, the editors and the reviewers. This article does not guarantee or endorse any products or any claims made by their manufacturers.

## Conflicts of Interest

The authors declare no conflicts of interest.

## Supporting information


**Figure S1.** The analytical process in this study.
**Figure S2.** Top markers in each cell clusters.
**Figure S3.** Identification of T‐cell sub‐populations
**Figure S4.** Prognostic model in TCGA COAD cohort.
**Figure S5.** Knock‐down MIF inhibits CRC cell growth and invasion. (A) The basal mRNA expression of MIF in six CRC cell lines was analysed using qRT‐PCR. The expression levels of MIF mRNA were normalised to the expression level of GAPDH. (B) The effectiveness of MIF knock‐down in HCT116 and Lovo cells was assessed by performing qRT‐PCR. (C) The cell viability of HCT116 and Lovo cells with down‐regulated MIF expression was assessed at 0, 24, 48 and 72 h. (D) The migratory and invasive capabilities of HCT116 and Lovo cells were compared using Trans well chambers. si‐RNA was employed to silence MIF in HCT116 CC cell lines. The bars represent the mean ± SD from three independent experiments. *p < 0.05; **p < 0.01.

## Data Availability

Additional data supporting the conclusions of this study can be accessed from the corresponding author upon a reasonable request.
