# Supplementary material for: Integration of Single‐Cell RNA Sequencing Data and Bulk Sequencing Data to Characterise the CD8+ T‐Cell Exhaustion Mediated Immune Microenvironment in CRC
Source: J Cell Mol Med. 2025 May 12;29(9):e70556. doi: 10.1111/jcmm.70556 (PMC12069026; doi:10.1111/jcmm.70556)
Supplement: Supplementary file 1 — Figure S1. The analytical process in this study. Figure S2. Top markers in each cell clusters. Figure S3. Identification of T‐cell sub‐populations Figure S4. Prognostic model in TCGA COAD cohort. Figure S5. Knock‐down MIF inhibits CRC cell growth and invasion. (A) The basal mRNA expression of MIF in six CRC cell lines was analysed using qRT‐PCR. The expression levels of MIF mRNA were normalised to the expression level of GAPDH. (B) The effectiveness of MIF knock‐down in HCT116 and Lovo cells was assessed by performing qRT‐PCR. (C) The cell viability of HCT116 and Lovo cells with down‐regulated MIF expression was assessed at 0, 24, 48 and 72 h. (D) The migratory and invasive capabilities of HCT116 and Lovo cells were compared using Trans well chambers. si‐RNA was employed to silence MIF in HCT116 CC cell lines. The bars represent the mean ± SD from three independent experiments. *p < 0.05; **p < 0.01. [file JCMM-29-e70556-s001.zip › Figure S1.pdf]

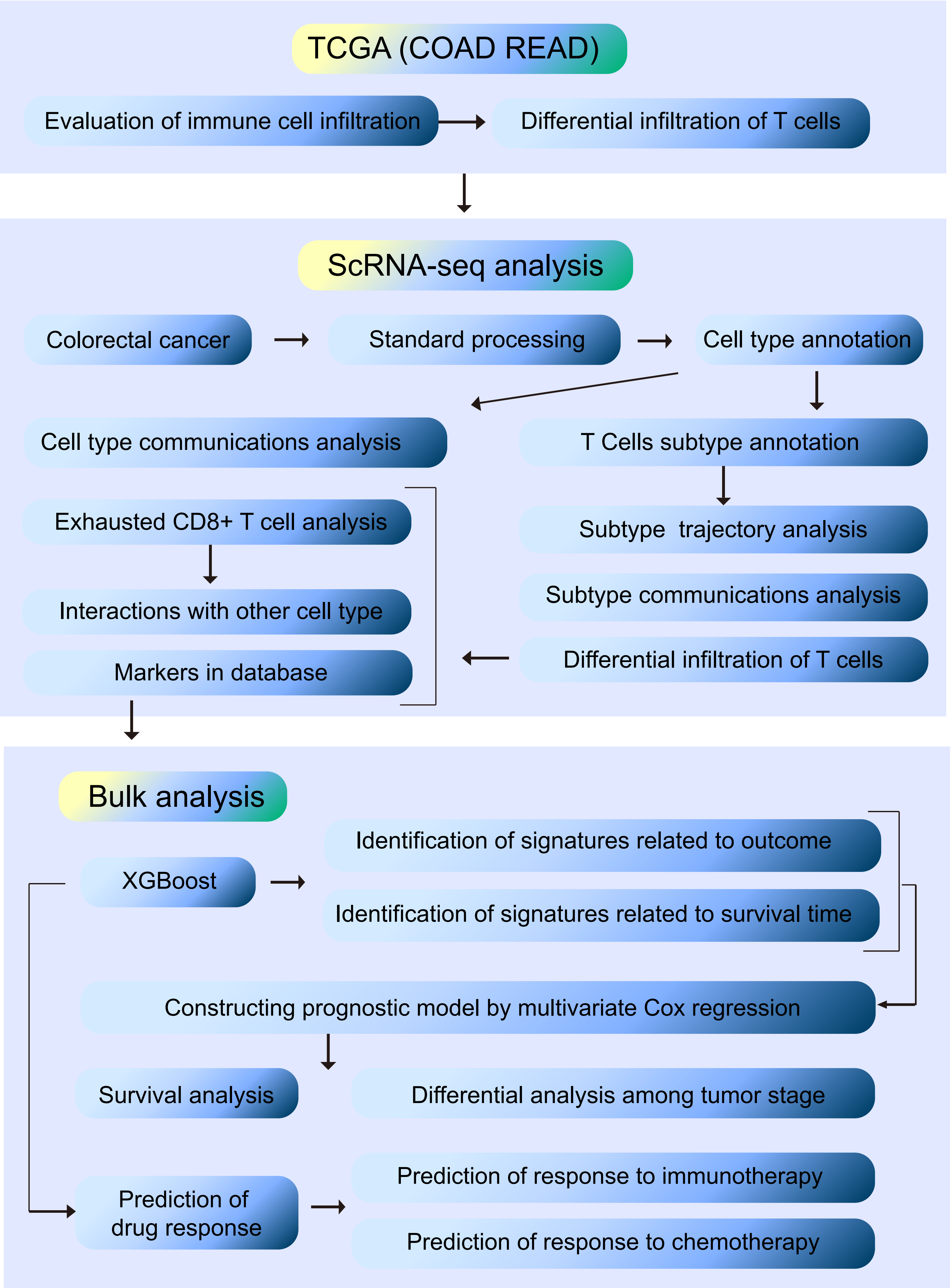

TCGA (COAD READ)
Evaluation of immune cell infiltration
Differential infiltration of T cells
ScRNA-seq analysis
Colorectal cancer
Standard processing
Cell type annotation
Cell type communications analysis
T Cells subtype annotation
Exhausted CD8+ T cell analysis
Subtype trajectory analysis
Interactions with other cell type
Subtype communications analysis
Markers in database
Differential infiltration of T cells
Bulk analysis
XGBoost
Identification of signatures related to outcome
Identification of signatures related to survival time
Constructing prognostic model by multivariate Cox regression
Survival analysis
Differential analysis among tumor stage
Prediction of drug response
Prediction of response to immunotherapy
Prediction of response to chemotherapy
