# Supplementary material for: Integration of Single‐Cell RNA Sequencing Data and Bulk Sequencing Data to Characterise the CD8+ T‐Cell Exhaustion Mediated Immune Microenvironment in CRC
Source: J Cell Mol Med. 2025 May 12;29(9):e70556. doi: 10.1111/jcmm.70556 (PMC12069026; doi:10.1111/jcmm.70556)
Supplement: Supplementary file 1 — Figure S1. The analytical process in this study. Figure S2. Top markers in each cell clusters. Figure S3. Identification of T‐cell sub‐populations Figure S4. Prognostic model in TCGA COAD cohort. Figure S5. Knock‐down MIF inhibits CRC cell growth and invasion. (A) The basal mRNA expression of MIF in six CRC cell lines was analysed using qRT‐PCR. The expression levels of MIF mRNA were normalised to the expression level of GAPDH. (B) The effectiveness of MIF knock‐down in HCT116 and Lovo cells was assessed by performing qRT‐PCR. (C) The cell viability of HCT116 and Lovo cells with down‐regulated MIF expression was assessed at 0, 24, 48 and 72 h. (D) The migratory and invasive capabilities of HCT116 and Lovo cells were compared using Trans well chambers. si‐RNA was employed to silence MIF in HCT116 CC cell lines. The bars represent the mean ± SD from three independent experiments. *p < 0.05; **p < 0.01. [file JCMM-29-e70556-s001.zip › Supplementary Information.docx]

**Supplementary Information**

**Supplementary Figures**

**Figure S1. The analytical process in this study.**

**Figure S2. Top markers in each cell clusters.**

**Figure S3. Identification of T cell subpopulations**

**Figure S4. Prognostic model in TCGA COAD cohort.**

**Figure S5. Knockdown MIF inhibits CRC cell growth and invasion**

**(A)** The basal mRNA expression of MIF in 6 CRC cell lines was analyzed using qRT-PCR. The expression levels of MIF mRNA were normalized to the expression level of GAPDH. **(B)** The effectiveness of MIF knockdown in HCT116 and Lovo cells was assessed by performing qRT-PCR. **(C)** The cell viability of HCT116 and Lovo cells with downregulated MIF expression was assessed at 0, 24, 48, and 72 hours. **(D)** The migratory and invasive capabilities of HCT116 and Lovo cells were compared using Trans well chambers. si-RNA was employed to silence MIF in HCT116 CC cell lines. The bars represent the mean ± SD from three independent experiments. **P* < 0.05; ** *P* < 0.01.
